# Study on the Inhibitory Effect of FOs on Advanced Glycation End Products (AGEs) Formation

**DOI:** 10.3390/foods15091610

**Published:** 2026-05-06

**Authors:** Yongmei Lyu, Haoxiang Wang, Xinying Ye, Zhihan Ge, Wanjie Mao, Zhipeng Cai, Xiaoyang Zhang, Wenlin Sun, Xiaohong Yu

**Affiliations:** 1 School of Marine and Bioengineering, Yancheng Institute of Technology, Yancheng 224051, China; lyu.yongmei@ycit.edu.cn (Y.L.); whx04137@163.com (H.W.); yexinying713@163.com (X.Y.); fbgphu74105246@163.com (Z.G.); maowanjie0522@163.com (W.M.); zhang.xiaoyang@ycit.edu.cn (X.Z.); swl2997553016@outlook.com (W.S.); 2 College of Food Science and Engineering, Jiangxi Agricultural University, Nanchang 330045, China; caizhipeng86@163.com

**Keywords:** feruloyl oligosaccharides (FOs), advanced glycation end products (AGEs), anti-glycation, inhibitors

## Abstract

This study focused on the inhibitory effects of wheat bran feruloyl oligosaccharides (FOs) on the formation of AGEs in three bovine serum albumin (BSA)-based non-enzymatic glycation models, namely BSA-fructose, BSA-methylglyoxal (MGO), and BSA-glyoxal (GO). In the BSA-fructose model, FOs at 0.25 mg/mL achieved a 62% inhibition rate of fructosamine, equivalent to approximately 78% of the activity of the positive control aminoguanidine (AG), and reduced fluorescent AGEs by over 50% on day 12. Additionally, FOs suppressed the accumulation of α-dicarbonyl compounds, key intermediates in the glycation pathway. In the BSA-MGO and BSA-GO system, the decreased fluorescence intensity of tryptophan residues indicated that FOs bound to BSA, inducing conformational changes in the protein microenvironment; this binding also inhibited protein carbonyl formation and the loss of thiol groups, thereby modulating the protein glycation process. Compared with their precursors (ferulic acid, FA; xylooligosaccharides, XOS), FOs exhibited comparable or even superior inhibitory activity against specific AGE subtypes, suggesting a synergistic effect between the feruloyl and oligosaccharide moieties. Sodium dodecyl sulfate-polyacrylamide gel electrophoresis (SDS-PAGE) revealed that FOs reduced the band intensity of 90 kDa AGEs in the glycation system, indicating the inhibition of protein-fructose cross-linking. Fluorescence spectroscopy confirmed that FOs dynamically quenched BSA with a single binding site, and thermodynamic calculations demonstrated that the binding was spontaneous (ΔG < 0), primarily driven by hydrogen bonds and van der Waals forces (ΔH < 0, ΔS < 0). This study systematically investigated the anti-glycation activities of FOs and their precursors. The findings demonstrate that FOs are promising natural glycation inhibitors and provide important theoretical and experimental support for related research. Furthermore, this study establish a basis for the green and high-value utilization of agricultural by-products like wheat bran.

## 1. Introduction

**Figure 1:**
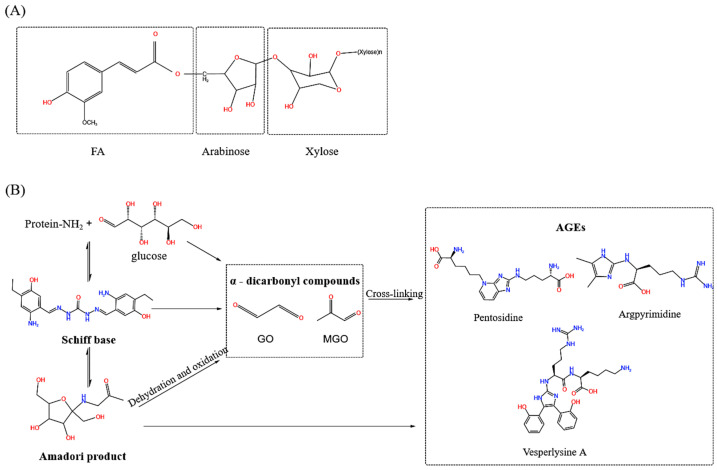
The structural formula of FOs (**A**) and the diagram of AGEs formation (**B**).

Feruloyl oligosaccharides (FOs) are a type of functional oligosaccharide whose molecular structure contains a ferulic acid (FA) group and an oligosaccharide chain, forming a complex connected via ester bonds (Figure 1A) [1,2]. This unique chemical structure endows FOs with multiple physiological functions integrating the properties of both FAs and Xylooligosaccharides (XOSs), including potent antioxidant and anti-glycation activities as well as regulatory effects on intestinal microbiota [3]. They are generally prepared from agricultural by-products such as corn bran [4,5], wheat bran [6], and rice bran [7,8]. Considering the abundance, low cost, and the potential value-added utilization of agricultural waste, wheat bran represents an abundant and readily available source of FOs. In 2010, FOs were granted GRAS (Generally Recognized as Safe) status by the FDA [9] and are used as food ingredients in baked goods, beverages, and dairy products to improve functional and nutritional properties, while increasing the added value of agricultural by-products [10].

Advanced Glycation End Products (AGEs) are a complex group of compounds formed via non-enzymatic glycation between reducing sugars and biological macromolecules such as proteins, lipids, or nucleic acids [11]. This reaction proceeds through three distinct stages: formation of an unstable Schiff base; its rearrangement to a relatively stable Amadori product; and subsequent dehydration, oxidation, and cross-linking to form AGEs (Figure 1B) [12]. Studies have confirmed that methylglyoxal (MGO) and 3-deoxyglucosone (3-DG) are key intermediates in AGE formation [11]. A high-glucose environment promotes MGO production via the glycolytic pathway, and MGO binds to lysine and arginine residues in proteins to form stable AGEs (e.g., MG-H1). Therefore, inhibition of the aldo-keto reductase (AKR) pathway involved in MGO metabolism has emerged as a novel target for intervening in AGE generation [12]. AGEs accumulation exerts diverse deleterious effects in vivo, mainly impairing the function of biological macromolecules and disrupting cellular and tissue structures. When AGEs bind to collagen, they induce loss of elasticity and cross-linking stiffening, leading to physiological changes such as skin aging and vascular stiffening [13]. Abnormal AGE accumulation also plays a critical role in the pathogenesis of neurodegenerative diseases by inducing inflammatory responses and exacerbating oxidative stress, thereby aggravating neuronal damage and promoting disease progression [14]. In addition, the rate of AGE formation is regulated by multiple factors: excessive free radical production during oxidation, monosaccharide autoxidation, and the presence of transition metal ions can all accelerate AGE generation, while an insufficient function of the body’s antioxidant system further promotes their accumulation [15].

Currently, strategies for intervening in AGE formation mainly focus on inhibiting key enzyme activities, scavenging free radicals, and alleviating oxidative stress. Some drugs and natural products can directly reduce AGE production by targeting and blocking key reactions in the AGE formation pathway, providing important insights for the screening of anti-AGE active substances and drug development [16]. Classic antioxidants such as vitamin C and vitamin E can indirectly inhibit AGE production via alternative pathways by scavenging excessive free radicals in the body and reducing systemic oxidative stress [17]. Chaiyana et al. reported that the favorable anti-AGE bioactivity of *Ocimum sanctum* Linn. makes it a natural skin anti-aging compounds candidate [18]. Wang et al. [19] reported that in the bovine serum albumin-glucose (BSA-Glu) non-enzymatic glycation model, ferulic acid and FOs quenched the fluorescence intensity of glycated BSA in a dose-dependent manner, with a maximum inhibition rate of 64.0%. Although preliminary evidence has confirmed the anti-glycation activity of FOs, their comprehensive inhibitory potential against AGEs still lacks systematic investigation. Systematic assessments of FOs in diverse glycation models—including BSA-fructose, BSA-methylglyoxal (MGO), and BSA-glyoxal (GO) systems—remain scarce. Furthermore, comparative studies on the antiglycation effects between FOs and their precursors (XOS and FA) are still insufficient.

In the present study, the antiglycation potential of FOs from wheat bran and their comparative inhibitory effects on AGE formation relative to their precursors were systematically investigated. Three bovine serum albumin (BSA)-based glycation models were established to assess the antiglycation activity of FOs. SDS-PAGE electrophoresis was employed to characterize the effects of FOs on AGE formation, while fluorescence spectroscopy was used to analyze their influence on BSA fluorescence spectra and interference with protein glycation. Collectively, this work systematically evaluates the antiglycation performance of FOs and preliminarily explores the underlying mechanism. These findings provide theoretical support for the development and application of FOs and their precursors, and facilitate the green, high-value utilization of wheat bran.

## 2. Materials and Methods

### 2.1. Materials

Aminoguanidine Hydrochloride was purchased from China National Pharmaceutical Group Chemical Reagent Co., Ltd. (Shanghai, China). Bovine serum albumin (BSA), nitroblue tetrazolium chloride (NBT), Girard’s reagent T (Girard-T), glyoxal, 2,4-dinitrophenylhydrazine (DNPH), guanidine hydrochloride (GdnHcl), Xylooligosaccharides (XOS), ferulaic acid (FA), aminoguanidine (AG), o-tolualdehyde (o-TA), potassium persulfate (KPS), ferric chloride (FeCl_3_), and 2-nitrobenzoic acid (DTNB), were all purchased from Shanghai Macklin Biochemical Co., Ltd (Shanghai, China). All reagents and chemicals used in this study were of the highest grade available. Wheat bran FOs were prepared by *Bacillus amyloliquefaciens IT-45* fermentation [20].

### 2.2. Establishment of a Protein-Fructose Simulated Glycation Reaction System

Equal volumes (1 mL each) of bovine serum albumin (BSA, 5 mg/mL), fructose (0.2 mol/L, as a glycation inducer), and different concentrations of FOs solutions were thoroughly mixed [21]. All reagents were dissolved in PBS buffer (0.2 M, pH 7.4). The positive control group contained aminoguanidine (AG) in the reaction system, while the blank control group contained only a mixture of BSA and fructose. Each mixture was incubated at 37 °C in a light-protected incubator for 12 days. Specific configurations of the test samples were as follows: FA group: 0.25 mg/mL ferulic acid solution; FF group: a composite solution containing 0.25 mg/mL FOs and 0.005 mg/mL FA; FO group: 0.25 mg/mL feruloyl oligosaccharide solution; XOS group: 0.25 mg/mL xylooligosaccharide solution; AG group: 0.25 mg/mL aminoguanidine solution. Each experiment was independently repeated three times to ensure data reliability.

### 2.3. Determination of Fructosamine Inhibition Rate

The BSA-Fru glycation reaction product solution and NBT solution (0.15 mM, pH 10.3, dissolved in 0.1 M sodium carbonate buffer) were mixed at equal volumes and incubated at 37 °C in the dark for 1 h [22]. A blank control with the same concentration of sodium carbonate buffer was used. Absorbance was measured at a wavelength of 530 nm, and the inhibition rate of fructosamine formation was calculated according to Formula (1).

(1) $$\mathrm{Fructosamine}\;\mathrm{inhibition}\;\mathrm{rate}\;(\%)=\frac{A_{0}-A}{A_{0}}\;\times \;100$$

Among them, A and A_0_ represent the absorbance of the sample group and the blank group at 530 nm, respectively.

### 2.4. Determination of the Inhibitory Capacity of α-Dicarbonyl Compounds

A pre-prepared mixed solution (containing 0.2 mL of 0.5 M Girard-T reagent and 3.4 mL of 0.5 M sodium formate buffer, pH 2.9) was sequentially added with 0.4 mL of BSA-fructose (Fru) glycation reaction solution [23], followed by vortexing. The mixture was then transferred to a 37 °C thermostatic environment and kept in the dark for 1 h to ensure sufficient reaction. A standard curve was established using gradient dilutions of a glyoxal standard. The absorbance of the reaction system was measured at 294 nm using a microplate reader. The inhibition rate of α-dicarbonyl compound formation was calculated according to Formula (1).

### 2.5. Determination of Fluorescent AGEs Inhibition Capacity

The BSA-Fru glycation reaction solution was taken, and its fluorescent signal intensities were detected under the conditions of four characteristic fluorescence wavelengths of AGEs (specific wavelength parameters are listed in Table 1) [24]. Based on the fluorescence intensity data, the inhibition rate of AGEs formation was calculated using Formula (1).

### 2.6. Establishment of the Protein-Methylglyoxal/Glyoxal (BSA-MGO/GO) Simulated Glycosylation Reaction System

Equal volumes of BSA solution (1 mg/mL), methylglyoxal/glyoxal (MGO/GO, 5 mM), and the sample solution were thoroughly mixed. The reaction mixture was dissolved in 0.2 M PBS buffer (pH 7.4) and incubated at 37 °C under constant temperature conditions for 72 h to generate the BSA-MGO/GO cross-linked products [25]. Under the same conditions, a BSA solution without MGO/GO was used as the blank control. Meanwhile, solutions containing neither the sample nor BSA and MGO/GO as the sample control, where the sample solution was prepared according to the previously mentioned protein-fructose model system. Each group was set up in triplicate.

### 2.7. Determination of Protein Carbonyl Content

The carbonyl content of BSA-MGO/GO glycated proteins was determined using the 2,4-dinitrophenylhydrazine (DNPH) colorimetric method [26]. One hundred microliters of various glycated protein samples were added separately into 400 μL DNPH solution (0.1%, *w*/*v*, dissolved in 2.5 M hydrochloric acid) and incubated at 25 °C for 1 h. Two milliliters of Trichloroacetic acid (TCA) solution (20%, *w*/*v*) were added to precipitate the proteins. Following centrifugation and removal of the supernatant, the precipitate was re-dissolved with a mixture of ethanol and ethyl acetate (1:1, *v*/*v*). After another centrifugation step, the remaining precipitate was re-dissolved in 250 μL guanidine hydrochloride (6 M), and the absorbance was measured at 360 nm. The carbonylation content in the glycated samples was calculated using the following Formula (2):

(2) $$n\;=\;\frac{OD600}{m\;\times \;\varepsilon \;\times \;d}$$

Among them, n: carbonyl content (nmol/mg pro); OD600: absorbance of the sample solution at 360 nm; m: sample concentration (mg/mL); ε: molar extinction coefficient 2.2 × 10^4^ L/(mol·cm); d: quartz cuvette thickness.

### 2.8. Determination of Free Thiol Content in Proteins

The free thiol content in glycated BSA-MGO/GO proteins was determined using the 2-nitrobenzoic acid (DTNB) method [27]. At room temperature, 20 μL of the glycated sample was mixed with 180 μL of DTNB in phosphate-buffered saline (PBS, 0.2 M, pH 7.4). The mixture was incubated at 37 °C for 15 min, and the absorbance value was measured at 412 nm. The free thiol content of proteins in different glycated samples (expressed as mM Cys) was calculated using a cysteine standard curve.

### 2.9. Determination of Protein Oxidation Products and Tryptophan

Fluorescence spectroscopy was employed for the quantitative analysis of changes in the microenvironment of tryptophan residues in proteins induced by BSA-MGO/GO glycation [28]. As shown in Table 2, the fluorescence intensity of BSA before and after glycation was determined, with the slit widths set at 2.5 nm and 1.0 nm, respectively.

### 2.10. SDS-Polyacrylamide Gel Electrophoresis (SDS-PAGE)

The regulatory effect of FOs on protein cross-linking structures in the BSA-Fru glycation reaction was evaluated using SDS-PAGE. Solutions required for the experiment (including separating gel, stacking gel, and electrophoresis buffer) were prepared according to the method described by Li et al. [29]. Specific configurations of the test samples were as follows: FA group: 0.25 mg/mL ferulic acid solution; FO group: 0.25 mg/mL feruloyl oligosaccharide solution; FF group: a composite solution containing 0.25 mg/mL FOs and 0.005 mg/mL FA; XOS group: 0.25 mg/mL xylooligosaccharide solution.

### 2.11. The Determination of the Effect of Wheat Bran FOs on BSA Fluorescence Spectra

0.9 mL of BSA and 0.1 mL of wheat bran FOs were dissolved in PBS buffer (0.2 mol/L, pH = 7.4), respectively. The two solutions were then mixed to obtain experimental samples. The BSA concentration was 0.2 mg/mL, while the final concentrations of wheat bran FOs were 0, 25, 50, 100, 150, 200, and 400 μg/mL. After incubating the samples at 4, 25, and 37 °C (277, 298, and 310 K) in the dark for 2 h, fluorescence spectra were used, with the excitation wavelength of 280 nm, an emission wavelength range of 300–400 nm, and a slit width of 3 nm. The Stern–Volmer equation can be used to determine the fluorescence quenching mechanism through the relevant parameter calculations [30], as shown in Equation (3):

(3) $$\frac{F0}{F}\;=\;1\;+\;\mathrm{KsvCq}\;=\;1\;+\;Kq\tau 0Cq$$

Note: In the equation, F0 and F represent the fluorescence intensities of bovine serum albumin samples treated with FOs at different temperatures. Ksv: Stern–Volmer quenching constant; Kq: quenching rate constant; Cq: concentration of the quencher; τ0: lifetime of the fluorescent molecule in bovine serum albumin (8–10 s). The values of Ksv and Kq can be determined based on the slope of the regression equation of F0/F versus Cq.

Furthermore, the number of binding sites (n) and the binding constant (Ka) are calculated using Equation (4):

(4) $$\mathrm{lg}\;\frac{F0-F}{F}=\mathrm{lg}\;\mathrm{Ka}\;+\;n\;\times \;\mathrm{lg}\;\mathrm{Cq}$$

The binding mode between FOs and BSA was further investigated through thermodynamic parameters. Fluorescence emission data at different temperatures were calculated using thermodynamic Equations (5) and (6):

(5) $$\ln\;\frac{K2}{K1}=\frac{\Delta H\;(\frac{1}{T1}-\frac{1}{T2})}{R}$$

(6) ΔG=ΔH−TΔS=−RTlnKa 

In the formula: K1, K2 are the binding constants at T1 (277 K), T2 (298 K), and T3 (310 K); R is the thermodynamic constant [8.314 J/(mol·K)]; ΔH is the enthalpy change (kJ/mol); ΔS is the entropy change [J/(mol·K)]; ΔG is the Gibbs free energy (kJ/mol).

### 2.12. Data Processing and Statistical Analysis

All experiments were conducted in triplicate, and data are expressed as mean ± standard deviation (n = 3). Graphs were generated using Origin version 2022. Data analysis was performed with SPSS 25.0 software. Data were analyzed using one-way analysis of variance (ANOVA) followed by Tukey’s post hoc test. In the figures, values marked with different lowercase letters indicate significant differences (*p* < 0.05).

## 3. Results and Discussion

### 3.1. Inhibitory Effect of FOs on the Formation of Fructosamine

**Figure 2:**
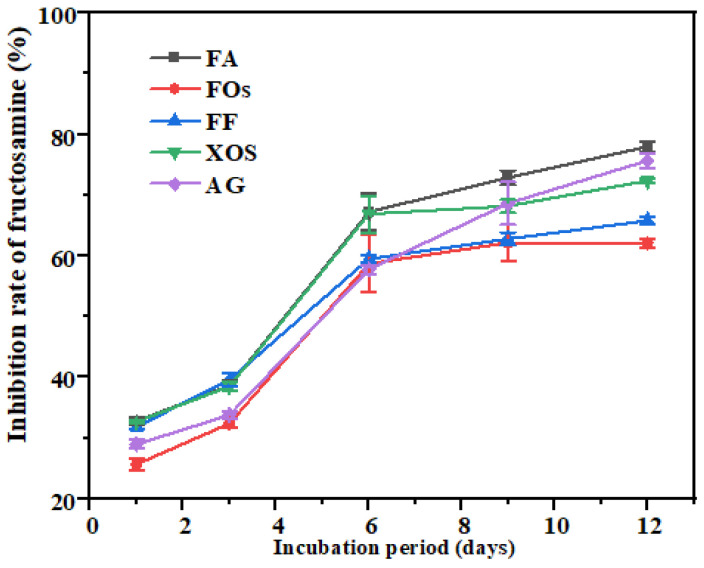
Fructosamine inhibition rate of FOs. Note: FA: Ferulic acid; FOs: Feruloyl oligosaccharides; FF: FA + FOs; XOSs: Xylooligosaccharides; AG: Aminoguanidine.

The BSA-Fru glycation model was employed to assess the anti-glycation capacity of FOs. The non-enzymatic glycation proceeds via three stages. In the early stage, reducing sugars react with protein amino groups to format labile Schiff bases (imine intermediates), which subsequently undergo Amadori rearrangement to yield stable fructosamine adducts [24]. As shown in Figure 2, the inhibitory effects of different samples on fructosamine formation displayed a three-stage dynamic pattern. Initial stage (0–2 days): A gradual increase in inhibition rate was observed, indicative of the accumulation phase of anti-glycation active ingredients. Rapid response phase (2–6 days): A sharp enhancement in inhibitory rate was detected, indicating the efficient binding of bioactive components to key molecular targets within the glycation pathway. Plateau phase (6–12 days): The rate of inhibition growth decelerated, with a steady state achieved at day 12, suggesting that the reaction system had reached a state of dynamic equilibrium. On day 12, FA exhibited a fructosamine inhibition rate of 78%, which surpassed that of the positive control aminoguanidine (AG). This finding is consistent with previous reports by Khan et al. [31]. Regarding the mechanism by which FA interferes with early glycation reactions: FOs alone achieved an inhibition rate of 62%. This may be attributed to the ferulic acid derivative moieties present in their molecular structure, which exert their inhibitory effects through competitive binding to protein amino groups [31]. Active substances disrupt the formation of Schiff bases either by scavenging α-dicarbonyl intermediates or by directly sequestering protein amino groups, thereby delaying the accumulation of Amadori products. This process is particularly prominent during the rapid response phase of the reaction.

### 3.2. Inhibitory Effect of FOs on the Formation of α-Dicarbonyl Compounds

**Figure 3:**
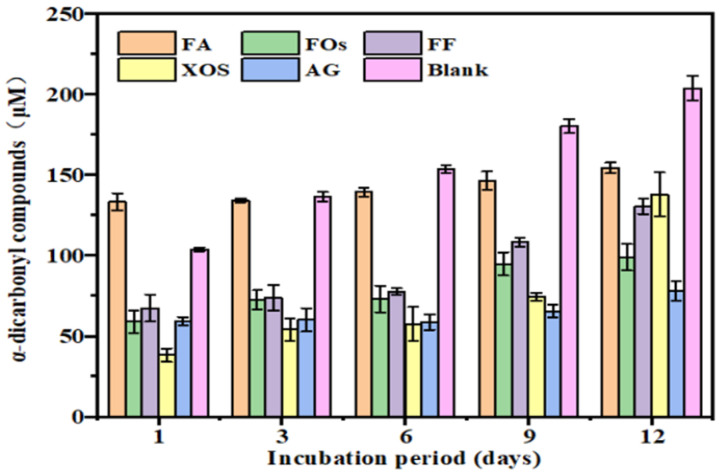
Inhibition rate of α-dicarbonyl compounds of FOs.

During the intermediate stage of non-enzymatic glycation, Amadori intermediates are converted into highly reactive α-dicarbonyl compounds (e.g., glyoxal and methylglyoxal) via oxidation, dehydration, and molecular rearrangements. These intermediates act as key precursors, mediating the formation of approximately 50% of AGEs [32]. As shown in Figure 3, α-dicarbonyl levels in all sample groups increased over time, indicating progressive glycation. However, on day 12, the levels of α-dicarbonyl concentrations in the FA, FO, FF, and XOS treatment groups were lower than in the blank control group (containing only BSA and fructose), indicating that these compounds suppressed α-dicarbonyl formation through distinct mechanisms.

XOS showed a time-dependent inhibitory pattern: XOS exhibited obvious inhibitory efficiency during the first three days (with the largest difference compared to the blank group), but its inhibitory rate decreased by day 12. This observation is consistent with the report by Singh et al. [28], indicating that XOS preferentially targets the early-stage pathway of α-dicarbonyl generation. FOs displayed a phased inhibitory effect: it exhibited potent activity in the initial six days, but α-dicarbonyl levels gradually rose as the reaction proceeded, approaching those in the XOS group by day 12. It is hypothesized that XOS moieties in FOs competitively scavenge reactive carbonyl groups, thus delaying α-dicarbonyl accumulation, yet exhibit lower long-term stability compared with FA.

### 3.3. Inhibitory Capacity of FOs on Fluorescent AGEs

**Figure 4:**
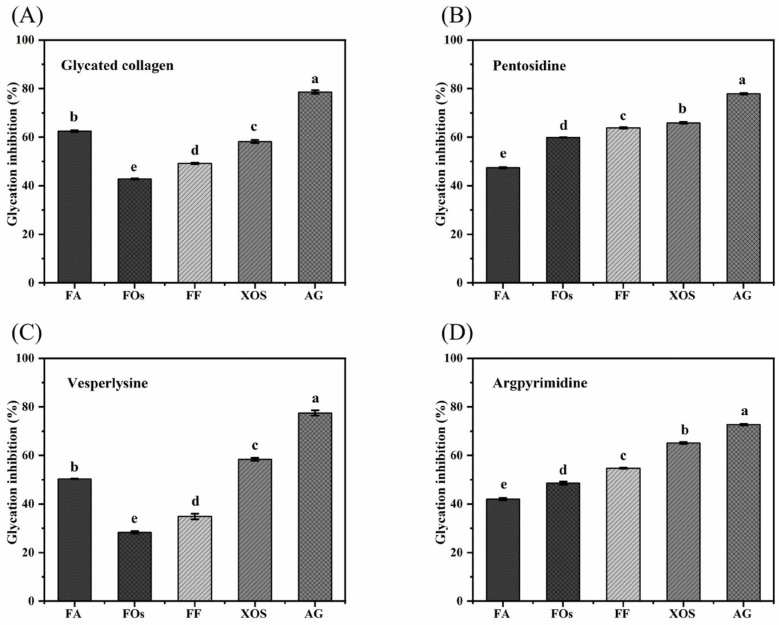
The inhibitory effect of each sample on AGEs. (**A**) Glycated collagen; (**B**) Pentosidine; (**C**) Vesperlysine; (**D**) Argpyrimidine. Note: Different lowercase letters indicate significant differences determined by one-way ANOVA (*p* < 0.05).

Abnormal accumulation of AGEs in vivo has been confirmed to be closely related to various aging-related diseases, including diabetic complications, neuronal damage in Alzheimer’s disease, and atherosclerotic plaque formation [33]. The underlying mechanisms involve enhanced oxidative stress, abnormal activation of receptor (RAGE) signaling pathways, and tissue dysfunction. Four typical AGEs (glycated collagen, Pentosidine, Vesperlysine, and Argpyrimidine) were detected by their characteristic fluorescence wavelengths as previously reported [24]. As shown in Figure 4, the inhibitory activities of all samples against four typical AGEs were weaker than those of the positive control aminoguanidine (AG), yet they exhibited subtype specificity. Notably, among the four tested AGE inhibitors, FF (FOs + trace amounts of FA) exhibited a slightly superior trend compared to FOs alone, suggesting that the supplementation of trace free FA could enhance the anti-glycation effects of FOs to some extent. This also validated the inhibitory role of the active group FA in FOs against AGE formation. As shown in Figure 4A,C, the inhibition rates of FA and XOS in the experimental samples were similar, both around 60%, indicating effective inhibition of glycated collagen and Vesperysine formation. In contrast, the inhibition rates of FOs and FF were slightly lower for these two types of AGEs, suggesting that the combination of ferulic acid and oligosaccharides in FOs may partially affect their inhibitory activity against glycated collagen and Vesperysine. In Figure 4B,D, the inhibition rates of FOs and FF against Pentosidine and Argpyrimidine both reached approximately 60%, higher than FA but close to XOS. This indicated that the FA and XOS groups may synergistically enhance FOs’ inhibitory effects on these two types of AGEs. Overall, FA and XOS demonstrated superior performance in inhibiting glycated collagen and Argpyrimidine, respectively. FOs exhibited consistent inhibitory effects on Pentosidine and Argpyrimidine, but the worst efficacy against Vesperysine. This indicated that FOs exhibited structural selectivity in inhibiting different AGEs formation, which might be attributed to their specific molecular conformations and the divergent formation pathways of various AGEs. Similar phenomena have been reported in other studies, such as the distinct inhibitory patterns and efficacy differences observed in Hsp-Cu(II) (naringenin-copper(II) complex), Hsp (naringenin), and AG across the three stages of non-enzymatic glycosylation reactions [34].

### 3.4. The Effects of FOs on the Oxidation of Tryptophan/Tyrosine During Protein Glycosylation

In the investigation of protein glycation processes, accurate measurement of tryptophan oxidation levels and protein oxidation products is crucial for evaluating the formation of AGEs [24]. As two key reactive carbonyl species, MGO and GO can react with amino groups of proteins to induce intermolecular cross-linking reactions of proteins, ultimately promoting the generation of AGEs [35]. This study employed the BSA-GO model to thoroughly investigate the effects of different samples on tryptophan oxidation and the overall protein oxidation status. As shown in Table 3, before the glycation reaction, the initial tryptophan fluorescence intensity of BSA was 572.97. After undergoing glycation treatment, the fluorescence intensity decreased to 258.89 in BSA-MGO model and 309.94 in BSA-GO model, respectively, directly indicating that approximately 46% of the tryptophan residues were consumed during the reaction.

During the protein glycation process, tryptophan residues are oxidatively modified into dityrosine and N′-formylkynurenine, while tyrosine residues are oxidatively modified into kynurenine [36,37]. The effects of different samples on the fluorescence intensities of dityrosine, N′-formylkynurenine, and kynurenine were presented in Table 3. FOs-treated samples exhibited reduced fluorescence intensity of N′-formylkynurenine, yet their inhibitory capacity was weaker than that of AG. Apart from AG, XOS more strongly inhibited the formation of dityrosine and kynurenine, whereas FA and FF showed only weak inhibitory effects on these two oxidation products. These results imply that the XOS fraction in FOs may protect tryptophan and tyrosine residues in BSA against oxidative damage. Moreover, in the BSA-MGO model, FA exerted a stronger inhibitory effect on N′-formylkynurenine formation than on dityrosine and kynurenine. By contrast, FF displayed potent inhibitory activity across the measured oxidation products in this model. These findings suggested that the FA component in FOs is more effective than the XOS component in suppressing tryptophan fluorescence.

### 3.5. The Effect of FOs on Carbonyl and Thiol Contents of Glycated-BSA

Protein glycation could lead to protein oxidation (increased carbonyl content) and reduced antioxidant capacity (decreased thiol group content) [38]. The effects of different treatments on protein carbonyl and thiol group contents are presented in Table 4. The initial carbonyl content of non-glycated BSA was 5.89 nmol/mg pro, whereas the carbonyl contents in glycated BSA (BSA-MGO/GO) increased significantly to 12.09 and 14.90 nmol/mg pro, respectively, indicating that glycation enhanced protein oxidation. BSA-MGO/GO systems treated with FA, FOs, FF, and XOS exhibited lower carbonyl contents than glycated BSA alone, suggesting that these treatments inhibited protein oxidation. Non-glycated BSA showed the highest thiol content (351 mM Cys), while glycated BSA displayed a pronounced reduction, with values of 43.5 and 16.4 mM Cys in BSA-MGO and BSA-GO, respectively, which was related to the consumption of thiol groups during glycation. Samples treated with FA, FOs, FF, and XOS all restored thiol group contents to varying degrees. Among them, FO treatment achieved the greatest recovery of thiol groups in both glycation models (except for the positive control AG), which may be attributed to its relatively strong antioxidant activity.

### 3.6. SDS-PAGE Analysis of BSA Treated with Wheat Bran-Derived FOs

**Figure 5:**
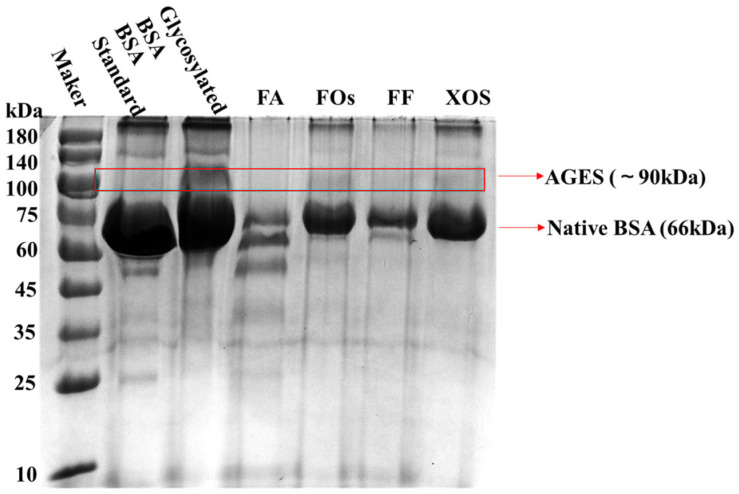
SDS-PAGE profile of the glycosylation of bovine serum albumin induced by wheat bran FOs and their precursors.

The impact of FOs on the molecular weight alterations of BSA during fructose-induced non-enzymatic glycation was systematically investigated by SDS-PAGE as previously described [39]. As illustrated in Figure 5, the BSA standard exhibited a distinct band at approximately 66 kDa, which is consistent with the known molecular weight of native BSA. In contrast, the BSA glycosylation reaction group (without any test samples) displayed, in addition to the intrinsic BSA band at 66 kDa, a relatively intense band at around 90 kDa; this band was identified as the characteristic band of non-enzymatically glycated BSA products. These observations indicated that during fructose-induced non-enzymatic glycation, BSA molecules undergo cross-linking reactions, leading to the formation of high-molecular-weight (HMW) adducts. Notably, the introduction of FA, FOs, FF, and XOS into the glycation reaction system resulted in an obvious reduction in the intensity of the 90 kDa band. This finding suggests that these samples can inhibit the formation of AGEs. This result is consistent with the findings reported by Akanksha et al. [28], who demonstrated that FOs are capable of competitively binding to glycation sites on proteins, reducing sugar-mediated protein cross-linking and subsequently reduces the formation of HMW glycation complexes.

### 3.7. Effect of Wheat Bran FOs on the Fluorescence Spectrum of BSA

**Figure 6:**
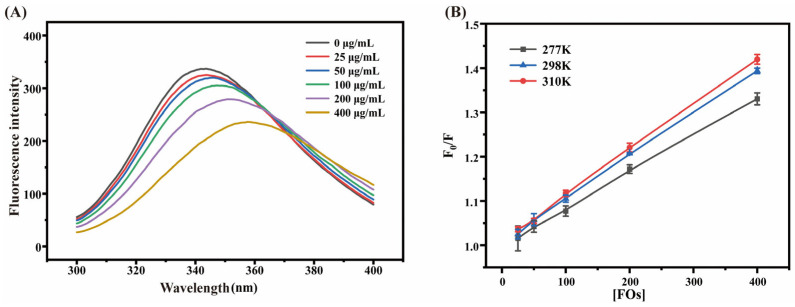
Fluorescence Spectroscopy Results of the Effect of FOs on the Glycosylation Modification of BSA. (**A**) Fluorescence spectra of different concentrations of wheat bran FOs and BSA at 280 nm under 298 K conditions; (**B**) Stern–Volmer curves of [FOs] and F0/F at different temperatures (277 K, 298 K, 310 K).

Two distinct mechanisms induce fluorescence quenching: the interaction between the quencher and the excited state of the fluorophore, or the quencher’s inhibition of the excited state formation of the fluorophore. It primarily encompasses two types, dynamic quenching and static quenching [40,41]. As shown in Figure 6A, at an excitation wavelength of λ_ex = 280 nm, the endogenous fluorescence spectrum of BSA changed with the concentration gradient of FOs. In the absence of FOs, the emission wavelength corresponding to the maximum fluorescence intensity of BSA (λ_max) was 343 nm. With increasing FOs concentration, the fluorescence intensity of BSA decreased, indicating that FOs might approach the tryptophan/tyrosine residues of BSA through intermolecular forces and quench its fluorescence. At the maximum concentration of400 μmol/mL FOs, the maximum fluorescence intensity of BSA shifted to an emission wavelength (λ_min) of 357 nm. The wavelength of the fluorescence peak exhibited a blue shift with a maximum shift value of 14 nm, suggesting that the conformation of BSA was altered under this fluorescence quenching condition. If the decay of fluorescence intensity exhibits a linear Stern–Volmer relationship with FOs concentration, it indicates predominant dynamic quenching; however, the presence of a saturation plateau, combined with shortened fluorescence lifetime, suggests that static quenching is dominant.

The Stern–Volmer curves of FOs at different temperatures were shown in Figure 6B. Combined with the calculated values listed in Table 5, it was found that the Ksv value of the BSA–FOs system decreased with increasing temperature, and the Kq value was higher than the maximum diffusion-controlled collision quenching constant of 2.0 × 10^10^ L·mol^−1^·s^−1^. These results indicated that FOs exerted dynamic quenching on BSA, and the quenching reaction in the BSA–FOs system belonged to dynamic quenching. This occurred because FOs interacted with BSA and quenched its intrinsic fluorescence. Therefore, the interaction mechanism between FOs and BSA can be regarded as dynamic quenching. Meanwhile, the binding site number (n) between FOs and BSA was approximately 1, suggesting the existence of only one binding site.

Based on the obtained binding constants and Ka values of FOs and BSA at three different temperatures, the values of ΔH, ΔS, and ΔG were calculated, and the resulting thermodynamic constants were listed in Table 6. The negative ΔG value indicated that the binding of FOs to BSA was a spontaneous process, according to the relationship between interaction force types and thermodynamic parameters summarized by Nan et al. [40]. Furthermore, the negative values of both ΔH and ΔS suggested that hydrogen bonding and van der Waals forces are primarily responsible for the interaction between FOs and BSA. Khan et al. [31] observed that the dynamic interaction between FA and BSA is spontaneous and associated with negative Gibbs free energy, where FA may inhibit the formation of glycation products and protein aggregation. Based on the above results, FOs interact with BSA via dynamic quenching, with hydrogen bonding and van der Waals forces as the dominant driving forces, and the interaction occurs spontaneously. This binding behavior further interferes with the glycation reaction between BSA and sugars, thereby inhibiting the formation of advanced glycation end products.

## 4. Conclusions

This study systematically evaluated the inhibitory effects of FOs on AGE formation and their key intermediate products by constructing non-enzymatic glycation reaction systems of BSA-fructose and BSA-MGO/GO. Experimental results indicated that at a concentration of 0.25 mg/mL, FOs exhibited anti-glycation activity in the BSA-fructose simulation system with the inhibition rate of 62%, corresponding to approximately 78% of the inhibitory activity of the positive control AG. FOs also reduced the formation of fluorescent AGEs by more than 50% under the same conditions. In the BSA-MGO/GO system, the addition of FOs further led to a decrease in the fluorescence intensity of BSA tryptophan residues, confirming that FOs could induce microenvironmental conformational changes by binding to proteins, thereby inhibiting carbonylation and thiol oxidation and subsequently interfering with the glycation reaction. SDS-PAGE analysis revealed that FOs could block fructose-mediated protein cross-linking reactions. Fluorescence spectroscopy analysis revealed that FOs induced dynamic quenching of BSA fluorescence, with a single binding site between FOs and BSA. The binding constant indicated moderate interaction strength. Thermodynamic parameters (ΔH < 0, ΔS < 0) further demonstrated that the binding of FOs to BSA was a spontaneous process (ΔG < 0), primarily driven by the synergistic effects of hydrogen bonding and van der Waals forces. Compared with their precursors, FOs exhibited comparable or stronger inhibitory activity against specific AGE subtypes, implying a synergistic effect between the feruloyl and oligosaccharide moieties. This finding provides a theoretical basis for the development of anti-glycation functional factors derived from natural products and laid a scientific foundation for the application of FOS in food processing and the intervention in chronic metabolic diseases. Future studies may focus on optimizing the structure-activity relationship of FOs and validating their in vivo anti-glycation efficacy.

## Figures and Tables

**Table 1:** Four characteristic fluorescence intensities of AGEs [24].

Wavelength	Glycated Collagen	Pentosidine	Vesperlysine	Argpyrimidine
Excitation Wavelength/nm	370	335	350	320
Transmission Wavelength/nm	440	385	405	380

**Table 2:** Fluorescence intensity of protein oxidation products measured by fluorescence.

Wavelength	Tryptophan	Dityrosine	N′-Formylkynurenine	Kynurenine
Excitation Wavelength (nm)	280	330	365	325
Emission Wavelength (nm)	350	415	480	434

**Table 3:** The effects of different treatment methods on the fluorescence intensity of four amino acids during BSA glycation.

Sample	Tryptophan	Dityrosine	N′-Formylkynurenine	Kynurenine
BSA	512.97 ±10.35 ^a^	4.51 ± 0.58 ^e^	3.45 ± 0.10 ^a^	3.42 ± 0.28
MGO + BSA	258.89 ± 8.65 ^b^	24.08 ± 2.16 ^bc^	2.96 ± 0.16 ^b^	14.32 ± 1.05 ^c^
MGO + BSA + FA	7.36 ± 0.76 ^e^	28.8 ± 1.23 ^b^	0.44 ± 0.06 ^g^	19.49 ± 1.30 ^a^
MGO + BSA + FOs	189.69 ± 5.68 ^c^	27.69 ± 2.61 ^b^	2.17 ± 0.24 ^c^	16.17 ± 0.75 ^b^
MGO + BSA + FF	27.85 ± 3.61 ^d^	33.87 ± 1.54 ^a^	0.91 ± 0.03 ^e^	17.53 ± 2.03 ^a^
MGO + BSA + XOS	288.64 ± 12.60 ^b^	9.48 ± 0.52 ^d^	1.15 ± 0.15 ^d^	5.38 ± 0.15 ^d^
MGO + BSA + AG	212.67 ± 12.86 ^c^	2.39 ± 0.30 ^f^	0.61 ± 0.07 ^f^	1.41 ± 0.08 ^e^
GO + BSA	309.94 ± 30.62 ^b^	21.30 ± 0.52 ^c^	2.31 ± 0.16 ^b^	8.04 ± 0.18 ^d^
GO + BSA + FA	5.46 ± 1.23 ^f^	30.20 ± 0.31 ^b^	0.79 ± 0.02 ^e^	16.39 ± 0.40 ^a^
GO + BSA + FOs	171.74 ± 10.32 ^c^	28.06 ± 1.26 ^b^	2.03 ± 0.24 ^c^	13.93 ± 1.03 ^c^
GO + BSA + FF	19.91 ± 2.65 ^e^	34.59 ± 2.85 ^a^	0.78 ± 0.12 ^e^	14.47 ± 0.52 ^b^
GO + BSA + XOS	289.99 ± 15.62 ^b^	10.11 ± 0.86 ^d^	1.47 ± 0.08 ^d^	3.10 ± 0.06 ^e^
GO + BSA + AG	96.76 ± 7.34 ^d^	2.98 ± 1.11 ^e^	0.31 ± 0.02 ^f^	2.93 ± 0.16 ^f^

Data are expressed as means ± SD (n = 3). Different superscript letters within the same column in the BSA-MGO and BSA-GO groups indicate significant differences determined by one-way ANOVA (*p* < 0.05).

**Table 4:** Effects of different components on protein carbonyl and thiol group contents.

Sample	Carbonyl Content (nmol/mg pro)		Thiol Groups (mM Cys)	
	BSA-MGO	BSA-GO	BSA-MGO	BSA-GO
BSA	5.89 ± 0.32 ^e^		351 ± 28 ^a^	
Glycated BSA	12.09 ± 0.09 ^a^	14.90 ± 0.09 ^a^	43.5 ± 1.3 ^f^	16.4 ± 0.2 ^g^
FA	11.64 ± 0.09 ^b^	10.79 ± 0.14 ^c^	121.4 ± 1.6 ^e^	24.3 ± 1.3 ^f^
FOs	9.67 ± 0.14 ^cd^	11.45 ± 0.18 ^b^	175.2 ± 8.8 ^c^	108.1 ± 0.4 ^c^
FF	9.32 ± 0.55 ^d^	10.57 ± 0.25 ^c^	124.6 ± 4.3 ^e^	74.3 ± 1.0 ^d^
XOS	9.73 ± 0.09 ^c^	10.58 ± 0.25 ^c^	137.2± 1.3 ^d^	29.5 ± 1.3 ^e^
AG	9.88 ± 0.05 ^c^	9.91 ± 0.09 ^d^	273.3 ± 0.8 ^b^	143.3 ± 0.6 ^b^

Data are expressed as means ± SD (n = 3). Different superscript letters within the same column indicate significant differences determined by one-way ANOVA (*p* < 0.05).

**Table 5:** Stern–Volmer quenching coefficient, binding constant, and binding site of the BSA-FOs system.

Temperature (K)	Ksv (×10^−4^ mg·mL^−1^)	Kq (×10^−12^ mg·mL^−1^·s^−1^)	Ka (mL·mg^−1^)	Rb	n
277	8.372	8.372	1.125	0.994	1.135
298	9.647	9.647	1.090	0.995	1.068
310	10.423	10.423	1.031	0.996	0.991

**Table 6:** Thermodynamic parameters of FOs/BSA.

	ΔH (×10^3^ k·J·mol^−1^)	ΔS (J·mol^−1^)	ΔG(×10^4^ k·J·mol^−1^)
277			−2.148
298	−2.935	−60.371	−2.303
310			−2.382

## Data Availability

The data presented in this study are available on request from the corresponding author.

## Abbreviations

The following abbreviations are used in this manuscript:

AGEs	Advanced glycation end products
AG	Aminoguanidine
BSA	Bovine serum albumin
FOs	Feruloyl oligosaccharides
DNPH	2,4-dinitrophenylhydrazine
DTNB	2-nitrobenzoic acid
FA	Feruloyl acid
XOS	Xylooligosaccharides
MGO	Methyl-glyoxal
NBT	Nitroblue tetrazolium chloride
GO	Glyoxal
SDS-PAGE	Sodium dodecyl sulfate-polyacrylamide gel electrophoresis
TCA	Trichloroacetic acid
